# Draft Genome Sequence of *Clostridium bifermentans* Strain WYM, a Promising Biohydrogen Producer Isolated from Landfill Leachate Sludge

**DOI:** 10.1128/genomeA.00077-14

**Published:** 2014-03-06

**Authors:** Y. M. Wong, J. C. Juan, H. M. Gan, C. M. Austin

**Affiliations:** a School of Science, Monash University Malaysia, Jalan Lagoon Selatan, Bandar Sunway, Malaysia; b Nanotechnology & Catalysis Research Centre (NANOCAT), University of Malaya, Kuala Lumpur, Malaysia

## Abstract

*Clostridium bifermentans* strain WYM is an effective biohydrogen producer isolated from landfill leachate sludge. Here, we present the assembly and annotation of its genome, which may provide further insights into the metabolic pathways involved in efficient biohydrogen production.

## GENOME ANNOUNCEMENT

*Clostridium bifermentans* was first identified by Tissier and Martelly (1). However, since its discovery, it has often been confused with *Clostridium sordellii* in terms of its taxonomic relationship because the strains share similar cultural, biochemical, morphological, and serological characteristics. Later, Nishida et al. (2) showed that *C. bifermentans* and *C. sordellii* have different sporulation behaviors, and Brooks et al. (3) differentiated these two species using gas chromatography. These results increased the confidence level for separating *C. bifermentans* and *C. sordellii* as different clostridial species. Today, with modern technologies, we can use whole-genome sequencing to further validate their taxonomic relationship.

*C. bifermentans* is a Gram-positive, anaerobic, and spore-forming bacterium (4, 5) that is commonly found in water, soil, sewage, and animal feces (6, 7). It is pathogenic and causes diseases such as bacteremia with metastatic osteomyelitis (8) and necrotizing pneumonia (9, 10). It ferments a wide range of carbohydrates, including glucose, fructose, maltose, glycerol, and sorbitol (4), and produces useful metabolites, such as acetate, lactate, ethanol, hydrogen, and carbon dioxide (11). Hence, it is a potential hydrogen producer.

*C. bifermentans* strain WYM was isolated from landfill leachate sludge. Sanitary landfills are very active in the biodegradation of organic waste. Therefore, the sludge originating from these landfills carries a similar microbial community and may contain efficient hydrogen producers. Sanitary landfills and their sludge are extreme environments that often have an imbalance of nutrients and fluctuating living conditions, such as pH and temperature. Hence, strain WYM, surviving in landfill leachate sludge, may have strong adaptability to harsh environments and may possess unique properties in the production of biohydrogen and biochemicals.

The genome sequencing of strain WYM was performed using the Illumina MiSeq benchtop sequencer (2 × 150-bp paired-end sequencing). The reads were trimmed and assembled *de novo* using the CLC Genomics Workbench 6.0 (CLC bio, Denmark). Multiple-genome alignment was conducted using Gegenees 2.0.3. The average similarities of the conserved core and the size of the core were set at 20% (12). The genome sequence was annotated with the Rapid Annotations using Subsystems Technology (RAST) server (13). RNAmmer 1.2 and tRNAscan-SE 1.21 were used to predict rRNAs and tRNAs, respectively (14, 15). Based on 16S rRNA analysis, strain WYM has 99 to 100% identity with many *C. bifermentans* strains, including strains E006 and E019. In addition, the heat plot from the multiple-genome alignment revealed that strain WYM shares up to 95% similarity with *C. bifermentans* ATCC 19299 AVNB01 and 88% with ATCC 638 AVNC01. These results suggest that strain WYM is a new strain of *C. bifermentans*. The draft genome sequence of strain WYM comprises 3,475,995 bases in 180 contigs. It has a G+C content of 28.02% and contains 3,380 genes, 5 rRNAs, and 51 tRNAs.

*C. bifermentans* WYM contains a dimeric [NiFe] hydrogenase that is regulated by the genes *hypA* and *hypB*. In addition, it contains genes encoding products such as acetate kinase, butyrate kinase, and ethanol dehydrogenase that are involved in the production of organic acids and solvents, including acetate, butyrate, and ethanol.

### Nucleotide sequence accession numbers.

This whole-genome shotgun project has been deposited at DDBJ/EMBL/GenBank under the accession no. AVSU00000000. The version described in this paper is version AVSU01000000.
